# Preparation and Application of *Clostridium perfringens* Alpha Toxin Nanobodies

**DOI:** 10.3390/vetsci11080381

**Published:** 2024-08-19

**Authors:** Qiong Jia, Hongrui Ren, Shuyin Zhang, Haoyu Yang, Shuaipeng Gao, Ruiwen Fan

**Affiliations:** College of Veterinary Medicine, Shanxi Agricultural University, Jinzhong 030801, China; b20201067@stu.sxau.edu.cn (Q.J.); 20232438@stu.sxau.edu.cn (H.R.); 20233846@stu.sxau.edu.cn (S.Z.); 20232434@stu.sxau.edu.cn (H.Y.); 20233919@stu.sxau.edu.cn (S.G.)

**Keywords:** *Clostridium perfringens*, nanobody, alpha toxin, neutralization

## Abstract

**Simple Summary:**

*Clostridium perfringens* is conditionally pathogenic, and its pathogenicity is caused by the toxins that it produces. Among these, the alpha toxin, produced by all subtypes of Clostridium perfringens, can cause enteritis or enterotoxemia in lambs, cattle, pigs, and horses, as well as traumatic clostridial myonecrosis in humans and animals, with high mortality. Therefore, the neutralization of the alpha toxin is crucial for the prevention and treatment of diseases caused by Clostridium perfringens. Here, the alpha toxin expressed in a prokaryotic expression vector was used to screen a nanobody against the alpha toxin from the natural nanobody library of an alpaca resource. The nanobody was characterized as having a half-life of 2.8 h, an affinity constant of 0.9 nmol/L, and good stability below 60 °C. The nanobody could effectively neutralize the phospholipase and hemolytic activity of the alpha toxin at a 15-fold ratio. In both Vero cells and model mice, the nanobody could neutralize the cytotoxicity of 2 × IC_50_ alpha toxin at certain concentrations, and it could rescue the cells and prevent the deaths of the mice. In conclusion, the specific nanobody against the alpha toxin could effectively neutralize the alpha toxin in Vero cells and mouse models, providing an anti-*Clostridium perfringens* toxin-related therapeutic product to inhibit the pathogenicity resulting from such toxins.

**Abstract:**

All subtypes of *Clostridium perfringens* (*C. perfringens*) produce the alpha toxin (CPA), which can cause enteritis or enterotoxemia in lambs, cattle, pigs, and horses, as well as traumatic clostridial myonecrosis in humans and animals. CPA acts on cell membranes, ultimately leading to endocytosis and cell death. Therefore, the neutralization of CPA is crucial for the prevention and treatment of diseases caused by *C. perfringens*. In this study, utilizing CPA as an antigen, a nanobody (CPA-VHH) with a half-life of 2.9 h, an affinity constant (KD) of 0.9 nmol/L, and good stability below 60 °C was prepared from a natural nanobody library from alpacas. The biological activity analysis of CPA-VHH revealed its ability to effectively neutralize the phospholipase and hemolytic activity of CPA at a 15-fold ratio. In Vero cells, 9.8 μg/mL CPA-VHH neutralized the cytotoxicity of CPA at two times the half-maximal inhibitory concentration (IC_50_). In a mouse model, 35.7 ng/g body weight (BW) of CPA-VHH neutralized 90% of the lethality caused by a 2× median lethal dose (LD_50_) of CPA. It was found that CPA-VHH protected 80% of mice within 30 min at 2 × LD_50_ CPA, but this dropped below 50% after 2 h and to 0% after 4 h. Rescue trials indicated that using CPA-VHH within 30 min post-infection with 2 × LD_50_ CPA achieved an 80% rescue rate, which decreased to 10% after 2 h. Furthermore, CPA-VHH effectively mitigated the reduction in the expression levels of zonula occludens-1 (ZO-1), Occludin, and Claudin-1, while also attenuating the upregulation of the pro-inflammatory cytokines interleukin-1β (IL-1β), interleukin-6 (IL-6), interleukin-7 (IL-7), interleukin-8 (IL-8), tumor necrosis factor α (TNF-α), and interferon-γ (IFN-γ) induced by CPA infection. Overall, this study has identified a specific nanobody, CPA-VHH, that effectively neutralizes CPA toxins in vitro and in animal models, providing a new tool for inhibiting the pathogenicity resulting from these toxins and laying an important foundation for the development of new anti-*C. perfringens* toxin-related therapeutic products.

## 1. Introduction

*Clostridium perfringens* (*C. perfringens*) is widely distributed in natural environments, especially in soil, sediments, and the intestinal tracts of both humans and animals [1]. Being a Gram-positive, anaerobic bacterium capable of forming endospores [2], *C. perfringens* is a conditional pathogen capable of causing various diseases, such as enteritis [3], gas gangrene [4], and necrotic enteritis [5]. These diseases affect a wide range of hosts, including livestock and poultry, resulting not only in animal welfare issues but also in significant economic losses [6], such as reduced productivity, increased treatment expenses, and animal mortality. Additionally, some diseases caused by *C. perfringens* can be transmitted to humans through the food chain, posing public health concerns.

The pathogenicity of *C. perfringens* is mainly due to the production of six exotoxins (α, β, ε, ι, CPE, and NetB) [4]. *C. perfringens* can be classified into seven subtypes (A-G) based on the types of toxins produced [7], each capable of producing the alpha toxin (CPA). CPA is a zinc metallophospholipase C, exhibiting phospholipase and sphingomyelinase activity [8], divided into two main structural domains, the catalytic N-terminal domain and the membrane-binding C-terminal domain. Both domains are immunogenic; however, only the C-terminal domain can stimulate a protective immune response [8,9]. The C-terminal region of the CPA protein shows a structural similarity to the C2-type lipid-binding domains of various eukaryotic proteins [10], such as synaptic adhesion proteins and pancreatic lipase, highlighting the importance of the CPA’s cell-membrane-binding region for its virulence and immunoprotective functions. CPA causes the lysis of host cells and tissue necrosis by cleaving the phospholipid bilayer structure of the cell membrane, thus disrupting the normal function of the cell membrane [9,10,11].

Antibodies, known for their high affinity and antigen specificity, have emerged as indispensable tools in diagnosing and treating numerous diseases. In addition to conventional antibodies, a distinct class of heavy-chain-only antibodies (HCAbs) has been identified in the serum of camelids and sharks [12,13]. HCAbs bind to antigens exclusively through a single variable domain, the variable domain of the heavy chain of HCAb (VHH), also recognized as single-domain antibodies (sdAbs) or nanobodies (Nbs). Nbs are only one-tenth of the size of traditional antibodies but retain a high degree of affinity and specificity [14]. These diminutive antibodies exhibit outstanding stability and solubility and great potential in various domains, such as medical diagnostics [15,16], disease treatment [17,18], drug delivery [14,19], and biotechnological research [15], owing to their ease of engineering and modification, as well as their exceptional penetrative capabilities.

Based on the advantages of nanobodies, we intended to construct a nanobody to neutralize the highly lethal toxin CPA to provide a new tool for CPA treatment. Here, CPA was used as an antigen to screen for CPA-specific nanobodies (CPA-VHH) from a natural nanobody library from alpacas, followed by an evaluation of its characteristics. To determine the potential function of CPA-VHH in neutralizing CPA, toxin neutralization assays—both in vitro using Vero cells and in vivo using mice—were performed to assess the biological efficacy of CPA-VHH.

## 2. Materials and Methods

### 2.1. Preparation of Recombinant CPA Protein

The CPA-flag plasmid was constructed based on the known CPA sequence from NCBI (GenBank: KY584046.1) by Sangon Biotech (Shanghai, China). The recombinant CPA plasmid was mixed with BL21 (DE3) competent cells, followed by heat shock for 45 s. A total of 1 mL of fresh LB medium was added, and the sample was shaken and cultured for 1 h. It was then transferred to 5 mL of LB medium supplemented with ampicillin for overnight culture. The following day, the culture was transferred at a 1:100 ratio into 1 L of medium and incubated at 37 °C with agitation at 200 rpm until it reached an OD_600_ of 0.8. Isopropyl-beta-D-thiogalactopyranoside (IPTG) was then added (final concentration of 0.2 mmol/L) to induce expression overnight at 15 ℃ with 120 rpm. Bacterial cells were collected through centrifugation at 7000× *g* for 10 min and lysis was performed using a sonicator (300 W, 30 min), followed by centrifugation at 20,000× *g* at 4 °C for 1 h. The supernatant was collected after centrifugation and filtered through a 0.22 μm filter for purification using the AKTA pure system (Cytiva, UT, USA).

### 2.2. Screening and Preparation of CPA Nanobodies

Immunotubes were coated with 20 μg/mL of CPA recombinant protein and incubated overnight at 4 °C, followed by blocking with MPBS (PBS + 2.5% skim milk) at room temperature for 2 h. The overnight-revived natural nanobody library from alpacas was precipitated with PEG-NaCl, and the phages were incubated in MPBS for 1 h. The blocked phages were added to the blocked immunotubes and incubated at 37 °C for 2 h, followed by washing the tubes with PBS. Phages were eluted using 2 mL of 100 mmol/L triethanolamine solution, followed by neutralization with 2 mL of Tris-HCl (1 mol/L, pH 7.4). The eluted phages were transferred to 16 mL of TG1 culture with an OD_600_ of 0.4 and incubated in a 37 °C water bath for 30 min. After the phage infection of the TG1 culture, the mixture was centrifuged, and the bacteria were resuspended in 2YT medium and plated on 2YTAG agar plates for overnight incubation at 30 °C to obtain a primary library. After three rounds of screening, 192 monoclonal colonies were picked from the quaternary library plates. The phage supernatant treated with the blocking solution was added to ELISA plates (Corning, SNY, New York, NY, USA) coated with CPA recombinant protein for specific binding, followed by incubation with HRP-conjugated anti-M13 phage antibody (1:15,000, Sinobiological, Beijing, China) as a secondary antibody for affinity identification and screening. The ELISA results of the clones were analyzed, with a ratio of the positive to negative value (P/N) ≥ 2.1 being the threshold for positive affinity. Clones with strong positive reactions and the correct sequence were named CPA-VHH. This was used to construct recombinant plasmids for induced expression and then for affinity chromatography purification.

### 2.3. Evaluation of CPA-VHH Characteristics

Temperature Stability Testing: Purified CPA-VHH underwent exposure to various temperatures (−20 °C, 4 °C, 37 °C, 60 °C) for different durations (0, 6, 12, 24, 48, 72 h). Initially, CPA-VHH was diluted at a ratio of 1:10, followed by a 2-fold serial dilution, to serve as the primary antibody. The temperature stability against CPA-VHH was evaluated using the ELISA method.

Half-Life Determination in vivo: A total of 20 8-week-old healthy male Balb/C mice were randomly divided into two groups. CPA-VHH was intravenously administered via the tail vein to ten mice, while an equivalent volume of PBS was administered to another ten mice as the controls. Blood samples were collected from the venous plexus behind the orbital socket at intervals of 1, 2, 4, 6, 12, and 24 h post-injection, and the serum was isolated. The serum was diluted 1:10 with PBS and used as the primary antibody to test the CPA-VHH levels in the serum of mice via anti-His-HRP through ELISA.

Affinity Determination with CPA: The affinity between CPA-VHH and the CPA recombinant protein was determined using a competitive ELISA method. The CPA recombinant protein was immobilized on an ELISA plate at 2 μg/mL (100 μL/well) and incubated overnight at 4 °C. The plate was subsequently blocked with a 3% BSA solution at 37 °C for 1 h. A series of gradient dilutions of the CPA recombinant protein (1.5^−1^ to 1.5^−20^ from the initial concentration of 40 nmol/L) were prepared, where it was mixed 1:1 with 50 μL of CPA-VHH (0.01 mg/mL) and incubated at 37 °C for 30 min. Then, the mixture was added to an ELISA plate to be incubated at 37 °C for 1 h. Mouse anti-His-HRP monoclonal antibody (1:15,000, CWBIO, Beijing, China) was subsequently added and the sample was incubated at 37 °C for 1 h. The OD_450_ was measured using a microplate reader (Bio-Rad, Hercules, CA, USA) after being developed with TMB for 15 min.

### 2.4. Neutralization of CPA-VHH on Phospholipase C and Erythrocyte Hemolyticity

Mixtures of CPA and CPA-VHH at ratios of 1:0, 1:1, 1:5, 1:10, 1:15, and 1:20 were incubated at 37 °C for 2 h. Then, the phospholipase C activity was assessed, a 50% egg yolk solution was diluted to 5% with PBS and centrifuged at 1000× *g* for 10 min, and the resulting supernatants were transferred to a 96-well plate (100 μL/well). Pre-prepared complexes of CPA and CPA-VHH were added to the wells, followed by incubation at 37 °C for 2 h. The OD_620_ was measured after incubation. The hemolytic activity was assessed using a freshly prepared 2% erythrocyte suspension (100 μL) in a centrifuge tube. After incubating the mixture of CPA and CPA-VHH at 37 °C for 2 h and centrifuging it at 1000× *g* for 5 min, 100 μL of the supernatant was transferred to a new microtiter plate, and the OD_545_ was measured.

### 2.5. Determination of the Half-Maximal Inhibitory Concentration (IC_50_) of CPA in Vero Cells

Vero cells stored in our laboratory were seeded at a density of 5 × 10^4^ cells/mL in 96-well plates and cultured in complete medium at 37 °C and 5% CO_2_ overnight. Following overnight incubation, the non-adherent cells were removed, and CPA was added to achieve a final concentration of 0, 0.1, 0.2, 0.6, 0.8, 1.0, 1.2, 1.4, 1.6, 1.8, and 2.0 μg/mL. After 12 h, CCK-8 solution (10 µL) was added to each well, and the cells were cultured for an additional 2 h. The OD_450_ was measured. The IC_50_ values were determined using GraphPad’s IC_50_ model.

### 2.6. Investigation of CPA-VHH’s Neutralizing Effect on CPA in Vero Cells

CPA at 2 × IC_50_ and CPA-VHH were mixed at varying ratios (1:0, 1:1, 1:5, 1:10, 1:15, 1:20) and incubated at 37 °C for 2 h. The control group was treated with various concentrations of CPA-VHH without CPA. Cells not exposed to CPA-VHH served as the blank control group. The CPA and CPA-VHH complex was added to Vero cells, and the samples were incubated at 37 °C with 5% CO_2_ for 12 h. Subsequently, 10 µL of CCK-8 reagent was added to each well and they were further incubated at 37 °C for 4 h. The OD_450_ was measured. The optimal effective concentration of CPA-VHH was denoted as m (VHH).

To assess the potential protective effects of CPA-VHH on Vero cells, a concentration of 2 × m (VHH) was initially administered to the cells. Subsequently, 2 × IC_50_ CPA was added at intervals of 0.5, 1, 2, 3, and 4 h, with an equivalent volume of PBS serving as the control. The cells were cultured at 37 °C with 5% CO_2_ for 12 h, and the cell viability was subsequently assessed using the CCK-8 assay.

### 2.7. Determination of Median Lethal Dose (LD_50_) for CPA

The LD_50_ values of mice injected with CPA via the tail vein were determined in this study. Six-week-old Balb/C mice were randomly divided into six groups, each consisting of 10 mice. Each mouse in each of the six groups was injected with 200 μL of CPA at varying doses of 0.3, 0.6, 0.9, 1.2, and 1.8 μg/g body weight (BW). The negative control group received injections of PBS. Subsequently, the number of surviving mice in each group was recorded within 24 h. The LD_50_ was calculated using the Bliss method via the SPSS 26 software.

### 2.8. Biological Effects of CPA-VHH in Mice Exposed to CPA 

To investigate the biological effects of CPA-VHH on CPA in mice, a total of 70 6-week-old Balb/C mice with a similar weight were randomly divided into 7 groups, with 5 males and 5 females in each group.

To assess the neutralizing effect of CPA-VHH on CPA in mice, 2 × LD_50_ CPA was mixed with CPA-VHH at varying ratios (1:0, 1:1, 1:3, 1:6, 1:9, 1:12, 1:15) and the mixtures were incubated at 37 °C for 2 h. Subsequently, these complexes were administered to different groups of mice via intravenous injection in the tail, with the negative control group receiving PBS injections. Each mouse was injected with a volume of 200 μL. The survival of the mice in each group was observed and recorded within a 12 h period, and survival curves were plotted. Additionally, the dosage of CPA-VHH resulting in the highest survival rate among the mice was designated as M (VHH).

To assess the protective effect of CPA-VHH against CPA infection in mice, an initial intravenous injection of 2 × M (VHH) was administered via the tail veins of the mice, followed by injections of 2 × LD_50_ CPA at different time intervals (15 min, 30 min, 1 h, 2 h, 3 h, 4 h). PBS was used as a negative control. The survival status of the mice was observed and recorded within a 12 h period.

To examine the therapeutic effect of CPA-VHH on CPA-infected mice, the mice were initially pre-injected with 2 × LD_50_ CPA to induce toxicity. Subsequently, the poisoned mice received an intravenous injection of 2 × M (VHH) via their tails at various intervals (15 min, 30 min, 1 h, 2 h, 3 h, 4 h). PBS was used as a negative control to replace CPA. The survival status of the mice was observed and recorded over a 12 h period.

To investigate the impact of CPA-VHH on genes and proteins associated with the intestinal mucosal barrier in CPA-infected mice, samples of duodenum were collected for quantitative real-time PCR and immunohistochemistry for mice that succumbed after receiving a dose of 2 × LD_50_ CPA and mice from the 30 min group that survived the rescue experiment following infection. 

### 2.9. Total RNA Isolation and Quantitative Real-Time PCR Analysis

Intestinal mucosa total RNA was extracted using the TRIzol reagent (Invitrogen, Carlsbad, CA, USA), following the manufacturer’s protocol, and treated with DNase I (Millipore, Burlington, MA, USA). To quantify the mRNA, 1 μg of total RNA was reverse-transcribed using a cDNA synthesis kit (TaKaRa, Dalian, China), following the manufacturer’s protocol, and qPCR was conducted with the SYBR Green PCR Master Mix (TaKaRa). The qPCR analysis was conducted on a 7500 Fast Real-Time PCR system (Thermo Fisher Scientific, Waltham, MA, USA). The primer sequences are provided in Table 1. The melting curves for each sample were analyzed to confirm the amplification specificity. The abundance of mRNA transcripts including zonula occludens-1(ZO-1), Occludin, and Claudin-1, as well as cytokines Iinterleukin-1β (IL-1β), interleukin-6 (IL-6), interleukin-7 (IL-7), interleukin-8 (IL-8), tumor necrosis factor α (TNF-α), and interferon-γ (IFN-γ), was quantified using the comparative threshold cycle method, which was normalized to β-actin.

### 2.10. Immunohistochemistry (IHC)

The mouse duodenum was fixed in Bouin’s solution, embedded in paraffin, and cut into 6 mm slices. After dewaxing and antigen repair, the paraffin sections of the duodenum were incubated in 3% H_2_O_2_ at room temperature for 20 min to remove endogenous peroxidase. Then, the sections were washed with PBS three times and blocked in 3% BSA 37 °C for 30 min. The sections were incubated in mouse anti-ZO-1 antibody (1:400), anti-Occludin antibody (1:400), or anti-Claudin-1 antibody (1:200) diluted in blocking solution overnight at 4 °C. After washing them in PBS, the sections were incubated with goat anti-mouse IgG HRP (1:200) at 37 °C for 30 min. After the residual secondary antibody was washed away with PBS, the color was developed with the DAB Kit and the sections were counterstained with hematoxylin. The sections were dehydrated and sealed in neutral resin. The results were observed via microscopy (Olympus, Shinjuku-ku, Japan).

### 2.11. Statistical Analysis

The half-life, affinity, IC_50_, and LD_50_ of CPA-VHH were analyzed using the corresponding data models in the SPSS 26 software(SPSS software, Chicago, IL, USA). The GraphPad 8 software (GraphPad Software; La Jolla, CA, USA) was used to perform a variance analysis of the mRNA expression via one-way ANOVA. *p* < 0.05 was considered to indicate a significant difference, and *p* < 0.01 was considered to indicate an extremely significant difference.

## 3. Results

### 3.1. Preparation of CPA Recombinant Protein and CPA-VHH

**Figure 1:**
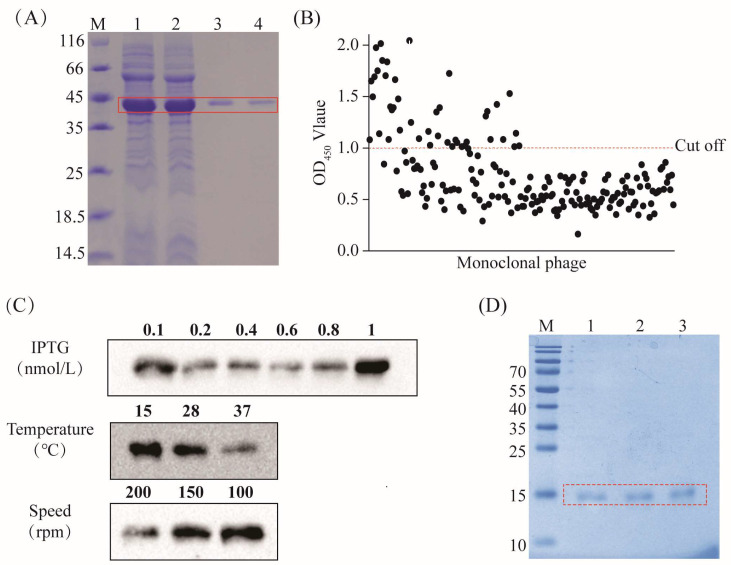
Screening and preparation of CPA-VHH. (**A**) Preparation of the CPA recombinant protein, highlighting the CPA protein within the red box; (**B**) phage ELISA screening, where the red dotted line is the OD_450_ value of 1.1; (**C**) optimization of CPA-VHH induction conditions (including the concentrations of IPTG, temperature, and speed); (**D**) preparation of CPA-VHH protein, with the CPA-VHH protein highlighted in the red box and samples 1–3 representing purified samples.

The recombinant plasmid was expressed in a prokaryotic system. After overnight induction at 15 °C, bacterial cells were collected, followed by sonication, and the resulting product was subjected to SDS-PAGE analysis. The results revealed the successful induction of the CPA recombinant plasmid, expressing a protein that was approximately 44.6 kDa in size. Purification using an anti-Flag tag pre-packed column yielded a nearly singular protein band of around 45 kDa with high purity (Figure 1A), facilitating the subsequent screening of the CPA-VHH.

Following four rounds of screening of the natural nanobody library with the CPA recombinant protein, 192 monoclonals were selected for phage ELISA testing at the conclusion of the fourth round. The clones with P/N ≥ 2.1, corresponding to an OD_450_ > 1.1, were deemed positive. A total of 49 clones met the standards (Figure 1B).

We selected the top 20 monoclonals exhibiting the strongest positive signals for sequencing. Based on the sequencing results, the recombinant plasmid encoding CPA-VHH was constructed and transformed into BL21 (DE3) to optimize the conditions for its induction. The results revealed that the optimal conditions for induction were achieved with a final IPTG concentration of 1 nmol/L and induction at 15 °C and 100 rpm overnight (Figure 1C). The purified samples were identified via SDS-PAGE, displaying a single band at 15 kDa with minimal impurities. The protein purity exceeded 85%, making the product suitable for further experiments (Figure 1D).

### 3.2. Evaluation of CPA-VHH Characteristics

**Figure 2:**
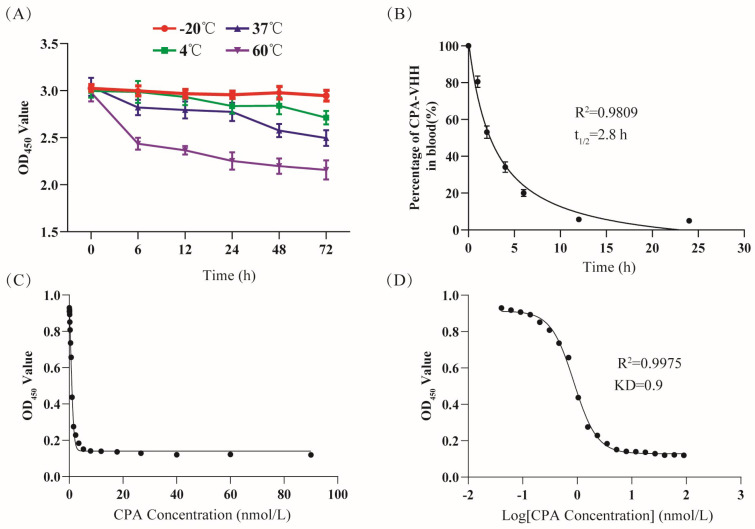
Evaluation of CPA-VHH characteristics. (**A**) Stability of CPA-VHH affected by temperature; (**B**) half-life of CPA-VHH; (**C**) fitting curve of OD_450_ and CPA-VHH concentration; (**D**) fitting curve of OD_450_ and log CPA-VHH concentration. Data represent mean ± standard error (*n* = 8).

Investigating the thermal stability of CPA-VHH involved its incubation under varied temperature conditions for different durations, with the subsequent assessment of its binding activity through ELISA. The findings revealed that the binding activity remained essentially unchanged for up to 72 h at −20 °C. With the increase in the temperature from 4 °C to 60 °C, there were decreases in the binding activity of CPA-VHH, with slight stability (Figure 2A).

The measurement of the CPA-VHH levels in mouse blood at different times facilitated the creation of a scatter plot, with the time on the *X*-axis and the percentage of CPA-VHH on the *Y*-axis, along with a fitted curve. The results showed that most of the samples were closely aligned with the curve, with an R^2^ value of 0.9809, which indicated a well-fitted curve. The analysis of the curve revealed a calculated half-life (t_1/2_) of 2.8 h (Figure 2B).

The CPA-VHH and CPA affinity was evaluated through the competitive ELISA method. The OD_450_ value reflected the content changes of CPA-VHH. Based on the use of the OD_450_ on the *Y*-axis and the CPA concentration and log CPA concentration on the *X*-axis, a fitting curve was created, with an R^2^ value of 0.9975. From the fitting curve, the KD value was calculated as 0.9 nmol/L (Figure 2C,D).

### 3.3. Neutralizing Effect of CPA-VHH on Phospholipase C and Erythrocyte Hemolyticity

**Figure 3:**
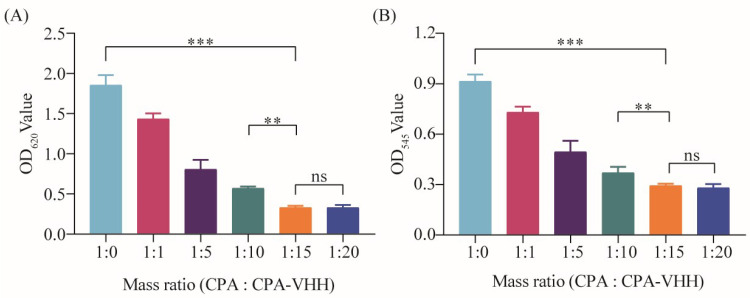
Neutralizing effect of CPA-VHH on phospholipase C and erythrocyte hemolysis. (**A**) Neutralizing effect of CPA-VHH on phospholipid hydrolysis; (**B**) neutralizing effect of CPA-VHH on erythrocyte hemolysis. Data represent mean ± standard error (*n* = 8) and were analyzed using one-way ANOVA, ns *p* > 0.05, ** *p* < 0.01, *** *p* < 0.001.

To investigate the neutralizing effect of CPA-VHH on the phospholipase activity of CPA, CPA was incubated with varying ratios of CPA-VHH before being introduced into a 5% egg yolk solution for phospholipase activity measurement. The results demonstrated that the egg yolk solution’s absorbance was the highest without CPA-VHH. In the other groups, the absorbance gradually decreased with the increasing CPA-VHH concentration. When the ratio of the egg yolk solution (including CPA) to CPA-VHH was 1:15 and 1:20, the lowest absorbance was observed, without significant differences (*p* > 0.05). However, a significant difference was observed between the 1:10 and 1:15 groups (*p* < 0.01). Compared to the 1:0 group, the neutralizing effect of CPA-VHH on the phospholipase activity was highly significant in the 1:15 group (*p* < 0.001, Figure 3A).

This research also investigated the neutralizing effect on the erythrocytes’ hemolytic activity by incubating CPA-VHH with CPA, followed by the addition of a 2% erythrocyte suspension. After incubation, neutralization was assessed by measuring the OD_545_. The results indicated a dose-dependent improvement in the neutralizing effect of CPA-VHH on the hemolytic activity of CPA, with a significant difference between the 1:0 and 1:15 groups (*p* < 0.001), as well as between the 1:10 and 1:15 groups (*p* < 0.01). However, increasing the dosage demonstrated no significant difference in the neutralization effect between the 1:15 and 1:20 groups (*p* > 0.05, Figure 3B).

### 3.4. Neutralizing Effect of CPA-VHH on CPA in Vero Cells

**Figure 4:**
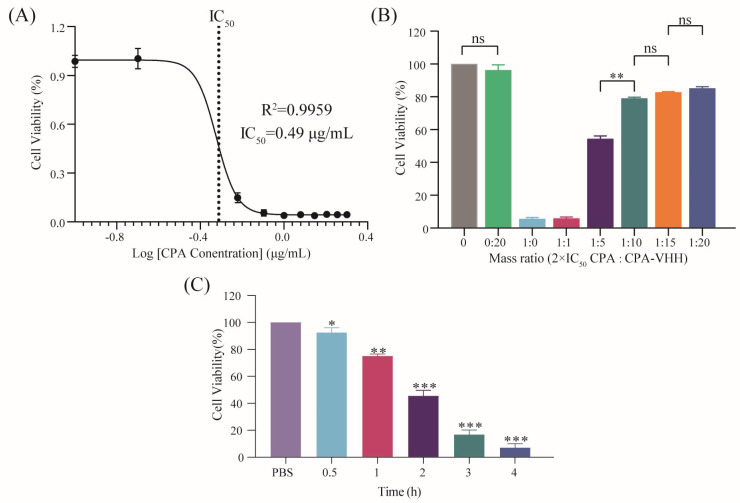
Neutralization of CPA toxicity by CPA-VHH in vitro. (**A**) Determination of IC_50_ in Vero cells by CPA; (**B**) neutralization of CPA toxicity by CPA-VHH in vitro; (**C**) protective effect of CPA-VHH on cells. Data represent mean ± standard error (*n* = 8) and were analyzed using one-way ANOVA, ns *p* > 0.05, * *p* < 0.01, ** *p* < 0.01, *** *p* < 0.001.

The IC_50_ of CPA on Vero cells was determined by adding various concentrations of CPA to the cells and culturing them for 12 h, followed by assessing the cell viability using the CCK-8 assay. The fitted curve, with the cell viability on the *Y*-axis and the log CPA concentration on the *X*-axis, yielded an R^2^ value of 0.9959. According to the IC_50_ model, the IC_50_ was calculated to be 0.49 μg/mL (Figure 4A).

The potential neutralization of CPA cytotoxicity by CPA-VHH was explored by incubating a mixture of 2 × IC_50_ CPA and CPA-VHH at various ratios for 2 h, prior to adding them to Vero cells, which were then cultured for 12 h. The cell viability was assessed using the CCK-8 assay. The results indicated that there was no significant difference in cell viability between the cells treated with CPA-VHH at a 0:20 ratio and the untreated control cells (*p* > 0.05), suggesting that CPA-VHH did not induce cytotoxicity in the Vero cells. Increasing amounts of CPA-VHH significantly improved the cell viability, indicating its neutralizing effect on CPA cytotoxicity. No significant differences were found among the 1:10, 1:15, and 1:20 groups (*p* > 0.05), but a significant difference existed between the 1:10 and 1:5 groups (*p* < 0.01). The optimal effective dose of CPA-VHH was determined to be 1:10, corresponding to m (VHH) = 9.8 μg/mL (Figure 4B).

The protective potential of CPA-VHH against CPA cytotoxicity in Vero cells was assessed by adding 2 × m(VHH) CPA-VHH to the cells, introducing 2 × IC_50_ CPA at different intervals, and finally incubating the cells for 12 h. The cell viability was then assessed using the CCK-8 assay. The results indicated that the cell viability gradually decreased as the time interval increased. Adding CPA at a 0.5 h interval resulted in a significant reduction in cell viability compared to the control group (*p* < 0.05). Intervals of 1 h or longer led to significantly different decreases in cell viability (*p* < 0.01, *p* < 0.001). These findings suggest that CPA-VHH provided protection to cells infected with CPA within a specific time range (Figure 4C). 

### 3.5. Effects of CPA-VHH in Terms of Neutralization of CPA and Prevention and Treatment of Infection with CPA in Mice

The LD_50_ of CPA was determined by monitoring the mice’s survival after the intravenous injection of various CPA doses. The survival rates decreased with increasing CPA dosages per gram of body weight. The LD_50_ was calculated as 1.2 ng/g using the Bliss method in SPSS.

**Figure 5:**
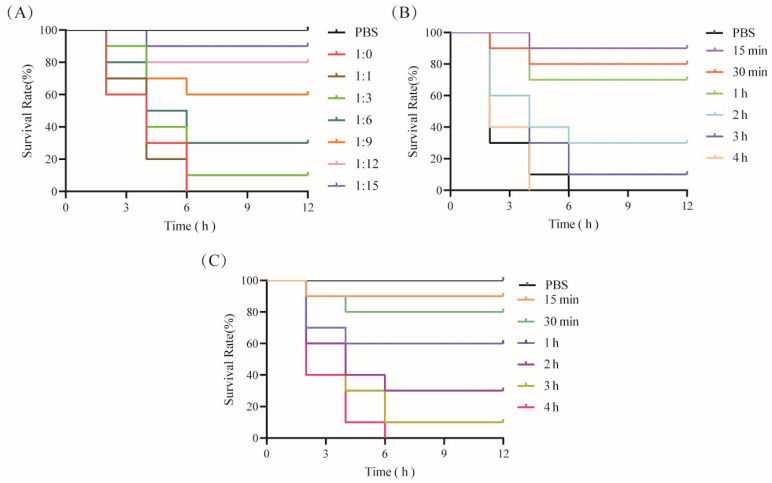
Neutralization of CPA toxicity by CPA-VHH in vivo. (**A**) CPA-VHH’s neutralization of CPA in mice; (**B**) preventative effect of CPA-VHH regarding CPA infection in mice; (**C**) CPA-VHH for treatment of mice infected with CPA. There were 10 mice in each group, namely 5 males and 5 females.

To assess the neutralizing effect of CPA-VHH on CPA in mice, 2 × LD_50_ CPA was mixed with CPA-VHH at varying ratios and incubated for 2 h before intravenous injection into the mice via the tail vein. The mice’s survival was monitored for 12 h. The results indicated that the mice injected with CPA alone succumbed within 6 h, while those injected with PBS or CPA-VHH survived at a rate of 100%. The mortality rates were 100% in the 1:1 group and 10% in the 1:3 group. The survival rates increased with higher ratios of CPA-VHH, with the optimal neutralizing dose observed at a 1:15 ratio (M(VHH) = 35.7 ng/g), achieving a 90% survival rate (Figure 5A).

To assess the protective effects of CPA-VHH against CPA, the mice were pre-injected with 2 × M (VHH), followed by the administration of 2 × LD_50_ CPA at varying intervals. The mice’s survival within 12 h was observed. The results showed that the mice injected solely with 2 × LD_50_ CPA died within 6 h. The mice injected with 2 × LD_50_ CPA 15 min after receiving 2 × M (VHH) had a 90% survival rate, which decreased to 80% when injected after 30 min. The survival rates dropped to less than 50% when 2 × LD_50_ CPA injection occurred more than 2 h after 2 × M (VHH) administration, and all mice died if the interval exceeded 4 h, resulting in 0% protection (Figure 5B). These findings indicate the time-dependent protection provided by CPA-VHH against CPA infection in mice.

The mice were initially injected with 2 × LD_50_ CPA to induce toxicity, followed by the subsequent administration of 2 × M (VHH) at varying intervals. The mice’s survival within 12 h was observed to assess the therapeutic effect of CPA-VHH on CPA-infected mice. The control group, injected solely with 2 × M (VHH) without prior CPA exposure, exhibited a 100% survival rate. Administering 2 × M (VHH) CPA-VHH at 15 min after the initial 2 × LD_50_ CPA injection rescued 90% of the mice, whereas a 30 min interval resulted in an 80% rescue rate. The therapeutic efficacy significantly declined if the interval exceeded 30 min, with a rescue rate of only 10% when the interval exceeded 2 h (Figure 5C).

### 3.6. Protective Effect of CPA-VHH on Intestinal Mucosa during CPA Infection

**Figure 6:**
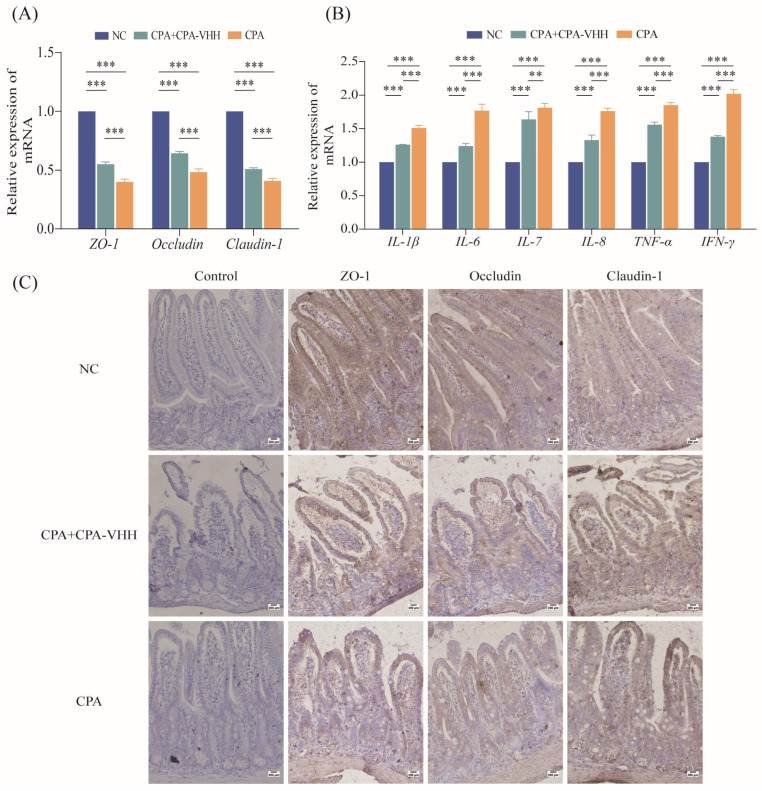
Protective effect of CPA-VHH on intestinal mucosa in CPA infection. (**A**) Effects of CPA-VHH on mRNA expression of ZO-1, Occludin, and Claudin-1 in intestinal mucosa of mice infected with CPA. (**B**) effects of CPA-VHH on expression of pro-inflammatory factors IL-1β, IL-6, IL-7, IL-8, TNF-α, and IFN-γ mRNA in intestinal mucosa of mice infected with CPA. Data represent mean ± standard error (*n* = 4) and were analyzed using one-way ANOVA, ** *p* < 0.01, *** *p* < 0.001. (**C**) effects of CPA-VHH on expression of tight junction proteins ZO-1, Occludin, and Claudin-1 in intestinal mucosa of mice infected with CPA.

To preliminarily explore the protective mechanism of CPA-VHH against CPA infection, the total RNA from the intestinal mucosae of negative mice (NC), of mice that died from a 2 × LD_50_ CPA challenge, and of mice that survived in the group that received the infection rescue treatment in 30 min were extracted to detect the expression of genes related to intestinal mucosal tight junction proteins and pro-inflammatory factors. The results indicated a significant decrease in the mRNA expression of tight junction proteins, including ZO-1, Occludin, and Claudin-1, in CPA-infected mice compared to negative mice (*p* < 0.001). The treatment of CPA-infected mice with CPA-VHH significantly increased the mRNA expression of these tight junction protein genes (*p* < 0.001), although they did not reach the levels observed in NC (Figure 6A). The mRNA expression of pro-inflammatory factors was also examined, which revealed a significant increase in the mRNA expression of IL-1β, IL-6, IL-7, IL-8, TNF-α, and IFN-γ in CPA-infected mice compared to the NC group (*p* < 0.001). Subsequent interventions with CPA-VHH resulted in significant decreases in IL-1β, IL-6, IL-7, IL-8, TNF-α, and IFN-γ (*p* < 0.01, *p* < 0.001, Figure 6B).

The IHC results showed that compared with normal mouse duodenal villi, the immune-positive signals of ZO-1, Occludin, and Claudin-1 in the duodenal mucosa were weakened to varying degrees after CPA infection. After CPA-VHH administration, the decreases in the positive signals of these tight proteins were alleviated to some extent (Figure 6C).

## 4. Discussion

In the early 1990s, a novel antibody isotype without light chain antibodies, named HcAbs, was discovered in the serum of camelid species [12] and later found in various cartilaginous fishes [20]. This novel antibody contains a single variable domain (VHH), which is the antigen-binding unit. The VHH domain retains its binding capacity, which makes it the smallest antigen-binding fragment [17,21]. In this study, CPA-VHH was purified and showed binding activity with CPA. 

Nanobodies exhibit remarkable stability, maintaining their integrity for several months at 4 °C [22]. At −20 °C, their shelf life can be further extended without affecting their antigen-binding functionality [22]. Incubation at 37 °C for several weeks does not significantly affect their stability [22]. Similarly, the stability of CPA-VHH was found to be good at −20 °C, although it decreased with prolonged exposure at 60 °C, but the binding activity was affected without significant differences. The extra disulfide bonds existing in the VHH domain are the main structures that maintain the stability of VHH [23].

Regarding the affinity between CPA-VHH and CPA, a competitive ELISA revealed a dissociation constant (KD) of 0.9 nmol/L, signifying the high affinity between the two entities. These findings propose CPA-VHH as a fundamental antibody for CPA detection, distinguished by its sensitivity and specificity. However, the small size and the absence of an Fc region in Nbs facilitate rapid renal clearance, which results in a short circulatory half-life of a few hours [24]. Thus, the fact that the half-life of CPA-VHH is short is understandable. However, it offers the possibility for the development of CPA-neutralizing drugs if it can be processed via bio-engineering methods.

*C. perfringens*, a Gram-positive, anaerobic bacterium, is pathogenic to both animals and humans [25]. It is widely distributed in the soil and the surrounding environment, secreting various toxins that cause intestinal diseases in both humans and livestock. All subtypes of *C. perfringens* produce CPA, a zinc-dependent enzyme, which exhibits phospholipase C and sphingomyelinase activity and damages cell membranes to cause hemolysis and necrosis, increasing the vascular permeability, and triggering platelet aggregation [4,8,9,26,27,28]. At present, there is no specific antibody drug for *C. perfringens*. Based on the binding activity, CPA-VHH could play a potential role in effectively inhibiting CPA-induced erythrocyte hemolysis, which could in turn offer the possibility to control enterotoxemia bleeding.

In Vero cells, the analysis of the protective efficacy of CPA-VHH revealed a decrease in its protective effect with longer intervals between CPA addition and CPA-VHH treatment. Thus, CPA-VHH effectively neutralized CPA’s cytotoxicity in vitro. Furthermore, CPA-VHH was demonstrated to possess 90% efficacy within 15 min of treatment of CPA-VHH in mouse models. However, beyond 2 h after the usage of CPA-VHH, the rescue efficacy dropped to 10%. The surviving mice demonstrated that CPA-VHH had stronger specific activity, which highlights the importance of its timely injection post-CPA infection to reduce mortality in livestock and poultry. The gradual decline in the therapeutic effect is primarily attributed to its short half-life. Enhancing its half-life through antibody engineering could potentially improve its therapeutic efficacy. Regarding the histopathological structure in mice treated with CPA, the intestinal mucosal barrier was disrupted. Tight junctions seal the paracellular space between cells, preventing the passage of large molecules [29,30]. In terms of the molecular mechanism of histopathology before and after the use of CPA-VHH, the proteins correlated to the tight junctions between cells are considered essential targets for maintaining the histological structure. ZO-1 is crucial for the formation of tight junctions in epithelial cells [31]. In the intestinal mucosae of mice, CPA stimulation led to a decrease in the mRNA expression levels of tight junction protein ZO-1. After the use of CPA-VHH, the decreased mRNA expression of ZO-1 was rescued, rendering the histological structure of the epithelial cells in the intestinal mucosa tight. CPA disrupted these tight junctions, which resulted in heightened mucosal permeability, facilitating the invasion of pathogens. Another two important proteins, Occludin and Claudin-1, form tight junctions in intestinal epithelial cells [32]. Occludin is essential in maintaining barrier function and plays a significant role in facilitating the translocation of large molecules from the intestine into the bloodstream [33]. Here, treatment with CPA-VHH significantly increased the expression of Occludin and Claudin-1. The results suggest that CPA-VHH could neutralize CPA and mitigate its detrimental effects on the physical barrier of the intestinal mucosa. 

Maintaining the equilibrium between pro-inflammatory and anti-inflammatory cytokines is vital for intestinal immune homeostasis [34]. IL-1β, IL-6, IL-8, and TNF-α are the key pro-inflammatory cytokines implicated in cytokine storms [35]. IL-6 is a multifunctional cytokine that is primarily responsible for mediating the acute-phase response, an innate immune mechanism activated by infection and inflammation [36]. IL-8, a CXC chemokine predominantly synthesized by macrophages, serves primarily as a chemotactic agent, thereby playing a significant role in inflammation [37]. TNF-α in mammals belongs to a group of NF-κB-activated signaling cytokines capable of inducing systemic inflammation [38,39]. It was reported that exposure to 1000 U/L of alpha toxin increased the expression of IL-6, IL-8, and TNF-α mRNA in the primary intestinal epithelial cells of chickens compared to the control group [40]. This research assessed the cytokine expression levels in the intestinal mucosal tissue of normal mice, CPA-challenged mice, and CPA-challenged mice treated with CPA-VHH using real-time quantitative PCR techniques. The results demonstrated that CPA administration resulted in the notable elevation of IL-1β, IL-6, IL-7, IL-8, and TNF-α cytokines; conversely, CPA-VHH effectively mitigated this increase. Hence, CPA-VHH exhibits anti-inflammatory properties, contributing to the maintenance of intestinal immune homeostasis.

## 5. Conclusions

In this study, a nanobody termed CPA-VHH was isolated from a naive nanobody library from alpacas using CPA as the antigen. CPA-VHH exhibited a half-life of 2.8 h, an affinity constant (KD) of 0.9 nmol/L, and demonstrated good thermal stability below 60 °C. The biological activity of CPA-VHH was assessed, confirming its capability to effectively neutralize CPA toxins both in vitro and in vivo. Although the results confirm that CPA-VHH is a new tool for mitigating the pathogenicity of this toxin, the therapeutic effect of CPA-VHH still needs to be studied in long-term animals. In conclusion, CPA-VHH holds significant promise for the development of neutralizing agents against *C. perfringens* infections.

## Figures and Tables

**Table 1:** Primer sequences.

Primer Name	Primer Sequences (5′-3′)
ZO-1-F	GCCGCTAAGAGCACAGCAA
ZO-1-R	GCCCTCCTTTTAACACATCAGA
Occludin-F	TTGAAAGTCCACCTCCTTACAGA
Occludin-R	CCGGATAAAAAGAGTACGCTGG
Claudin-1-F	GCCTTGATGGTAATTGGCATCC
Claudin-1-R	GGCCACTAATGTCGCCAGAC
IL-1β-F	TTCAGGCAGGCAGTATCACTC
IL-1β-R	GAAGGTCCACGGGAAAGACAC
IL-6-F	TCCAGTTGCCTTCTTGGGAC
IL-6-R	GACAGGTCTGTTGGGAGTGG
IL-7-F	TTCCTCCACTGATCCTTGTTCT
IL-7-R	AGCAGCTTCCTTTGTATCATCAC
IL-8-F	ATGCCCTCTATTCTGCCAGAT
IL-8-R	GTGCTCCGGTTGTATAAGATGAC
TNF-α-F	CCCTCACACTCAGATCATCTTCT
TNF-α-R	GCTACGACGTGGGCTACAG
INF-γ-F	ATGAACGCTACACACTGCATC
INF-γ-R	CCATCCTTTTGCCAGTTCCTC
β-actin-F	TTGCTGACAGGATGCAGAAG
β-actin-R	ACATCTGCTGGAAGGTGGAC

## Data Availability

The original contributions presented in this study are included in the article/Supplementary Materials; further inquiries can be directed to the corresponding author/s.

## Supplementary Materials

The following supporting information can be downloaded at: https://www.mdpi.com/article/10.3390/vetsci11080381/s1, Figure S1: Construction map of CPA prokaryotic expression plasmid.
