# Supplementary material for: Preparation and Application of Clostridium perfringens Alpha Toxin Nanobodies
Source: Vet Sci. 2024 Aug 19;11(8):381. doi: 10.3390/vetsci11080381 (PMC11360521; doi:10.3390/vetsci11080381)
Supplement: Supplementary file 1 [file vetsci-3148888-supplementary.pdf › vetsci-3148888-supplementary.pdf]

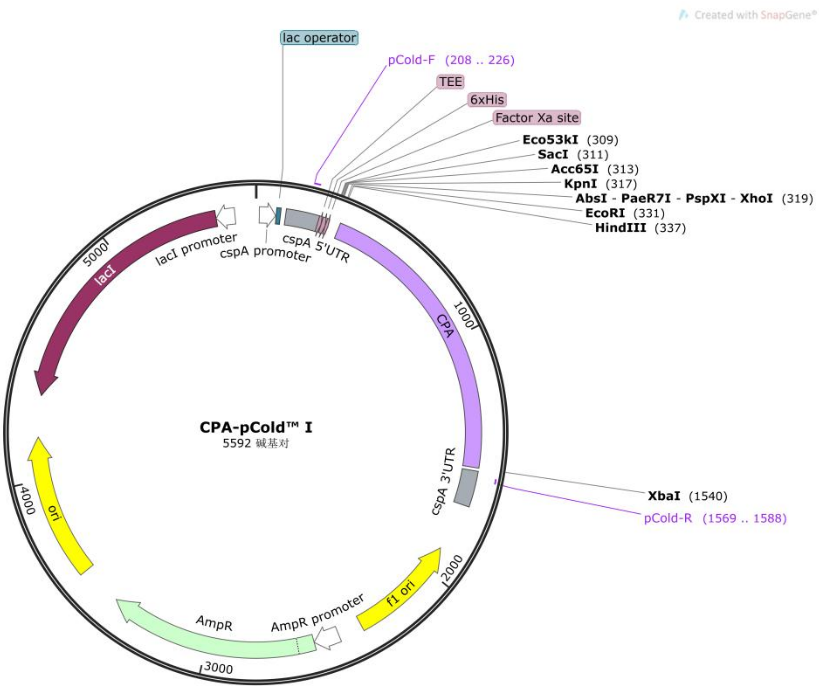

Figure S1. Construction map of CPA prokaryotic expression plasmid. The purple color in the map is the CPA sequence, which was inserted between the HindIII and XbaI restriction sites on the pcoldI plasmid.
